# Prion Domain of Yeast Ure2 Protein Adopts a Completely Disordered Structure: A Solid-Support EPR Study

**DOI:** 10.1371/journal.pone.0047248

**Published:** 2012-10-16

**Authors:** Sam Ngo, Vicky Chiang, Elaine Ho, Linh Le, Zhefeng Guo

**Affiliations:** Department of Neurology, Brain Research Institute, Molecular Biology Institute, University of California Los Angeles, Los Angeles, California, United States of America; Universitat Autònoma de Barcelona, Spain

## Abstract

Amyloid fibril formation is associated with a range of neurodegenerative diseases in humans, including Alzheimer’s, Parkinson’s, and prion diseases. In yeast, amyloid underlies several non-Mendelian phenotypes referred to as yeast prions. Mechanism of amyloid formation is critical for a complete understanding of the yeast prion phenomenon and human amyloid-related diseases. Ure2 protein is the basis of yeast prion [URE3]. The Ure2p prion domain is largely disordered. Residual structures, if any, in the disordered region may play an important role in the aggregation process. Studies of Ure2p prion domain are complicated by its high aggregation propensity, which results in a mixture of monomer and aggregates in solution. Previously we have developed a solid-support electron paramagnetic resonance (EPR) approach to address this problem and have identified a structured state for the Alzheimer’s amyloid-β monomer. Here we use solid-support EPR to study the structure of Ure2p prion domain. EPR spectra of Ure2p prion domain with spin labels at every fifth residue from position 10 to position 75 show similar residue mobility profile for denaturing and native buffers after accounting for the effect of solution viscosity. These results suggest that Ure2p prion domain adopts a completely disordered structure in the native buffer. A completely disordered Ure2p prion domain implies that the amyloid formation of Ure2p, and likely other Q/N-rich yeast prion proteins, is primarily driven by inter-molecular interactions.

## Introduction

Formation of amyloid fibrils and oligomeric intermediates is associated with a wide range of human disorders, including Alzheimer’s disease, prion diseases, and type II diabetes [1]. The amyloid fibrils involved in different diseases share common characteristics such as cross-β X-ray diffraction pattern, and binding to amyloid-specific dyes thioflavin T and Congo red, although different amyloid proteins are distinct in their amino acid sequences and native structures. These amyloid proteins can be divided into two groups: one group with a folded structure in their native soluble state, and the other group that is intrinsically disordered. For the intrinsically disordered proteins, there is strong interest in identifying residual structures. As protein folding studies have shown, residual structures in the unfolded state may be the initiating structure for the folded state [2], [3]. The residual structures in disordered amyloid proteins may play a critical role in nucleation of amyloid fibrils. To achieve this goal, one needs methods that are able to identify residual structures, preferably at residue level. Electron paramagnetic resonance (EPR) spectroscopy is a sensitive technique to study protein structure and dynamics, and continuous-wave EPR lineshape is sensitive on the picosecond to nanosecond timescale [4]. Structural transition from a denatured state to a structured state in proteins results in changes in backbone dynamics on the picosecond to nanosecond timescale, allowing EPR detection of residual structures [5], [6]. One example of such study is the binding-induced protein folding within the intrinsically disordered domain of the measles virus nucleoprotein, in which random coil to helix transition and a transiently populated folded state were detected with EPR [7]. We also show that a structured state of amyloid-β (Aβ) monomer can be detected by EPR [8].

One complication for studying the residual structure of intrinsically disordered amyloid proteins is that these proteins are aggregation-prone, resulting in the rapid formation of a mixture of monomers and aggregated species. When monomers and aggregates coexist in the sample, it is difficult to distinguish the residual structure in the monomer from the aggregate. To address this problem, we have developed a solid-support EPR approach [8], in which the protein is tethered on a solid support to prevent protein aggregation. This approach allows the use of high protein concentration, normal temperature, and near physiological buffer conditions without aggregation-inhibiting co-solvents. We have applied this approach to study the monomer structure of Aβ protein involved in Alzheimer’s disease, and show that the intrinsically disordered Aβ40 protein adopts both a disordered state and a structured state [8]. Here we apply this approach to study the prion domain of yeast prion protein Ure2.

**Figure 1:**
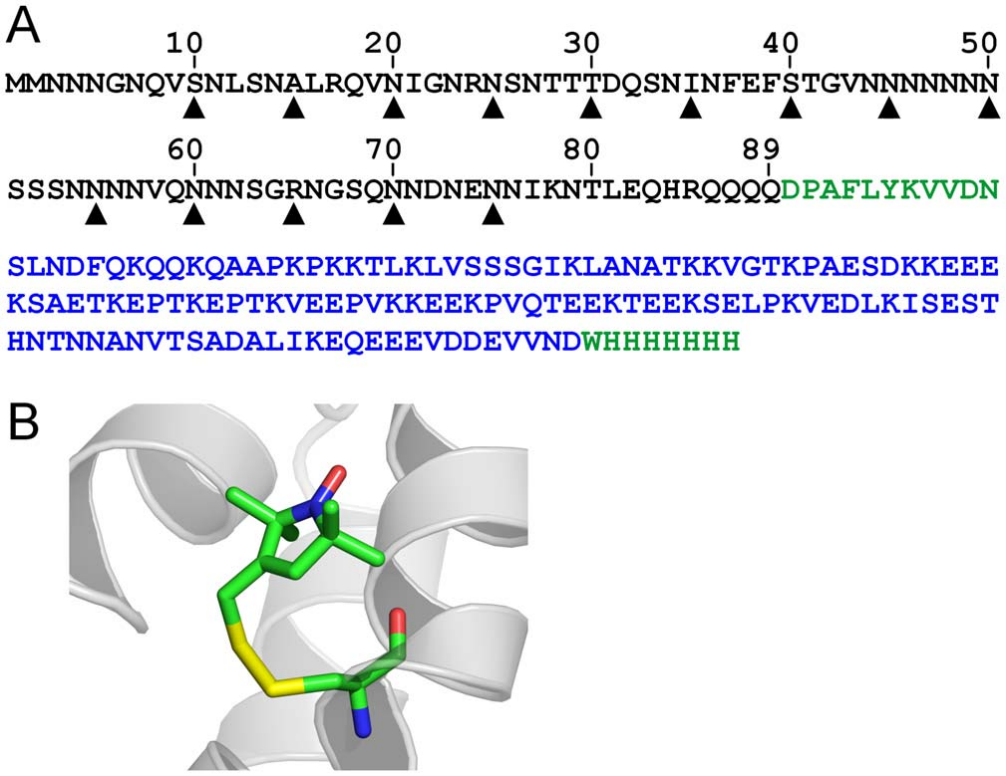
Spin labeling of Ure2p_1–89_-M. (A) Sequence of Ure2p prion domain construct with positions for spin labeling indicated by arrow heads. The amino acid sequences of Ure2p prion domain, Sup35p M domain, and other linker/tag regions are shown in black, blue, and green, respectively. (B) A stick model of spin label R1 in the crystal structure of spin-labeled T4 lysozyme (PDB entry 2IGC).

Ure2 is the protein whose aggregated form is responsible for the prion [URE3] in yeast *Saccharomyces cerevisiae*. The normal function of Ure2 protein is to suppress the protein expression involved in the uptake of poor nitrogen sources when a good nitrogen source is present. At the prion state [URE3], Ure2 protein is sequestered in the aggregated form, allowing the expression of nitrogen catabolism genes for poor nitrogen sources even in the presence of a good nitrogen source. The full-length Ure2 protein is 354-residue long, and consists of an N-terminal prion domain (∼90 residues) and a C-terminal functional domain [9], [10]. The C-terminal domain is necessary and sufficient for Ure2p’s cellular function. The N-terminal prion domain is required for the [URE3] prion phenotype. The structure of the C-terminal domain has been solved using X-ray crystallography [11], [12] and it has similarity to glutathione transferases. The overall architecture of Ure2p fibrils has been determined to contain an amyloid core formed by the prion domain, while the C-terminal domain retains its native structure in the fibril [13], [14], [15]. In its native state, the Ure2p prion domain is sensitive to protease digestion, suggesting that it does not have a folded structure [16]. Circular dichroism studies support this view [17]. Solution NMR studies on full-length Ure2p have shown that most residues in the prion domain are flexible, suggesting that the prion domain is largely unstructured [18].

**Figure 2:**
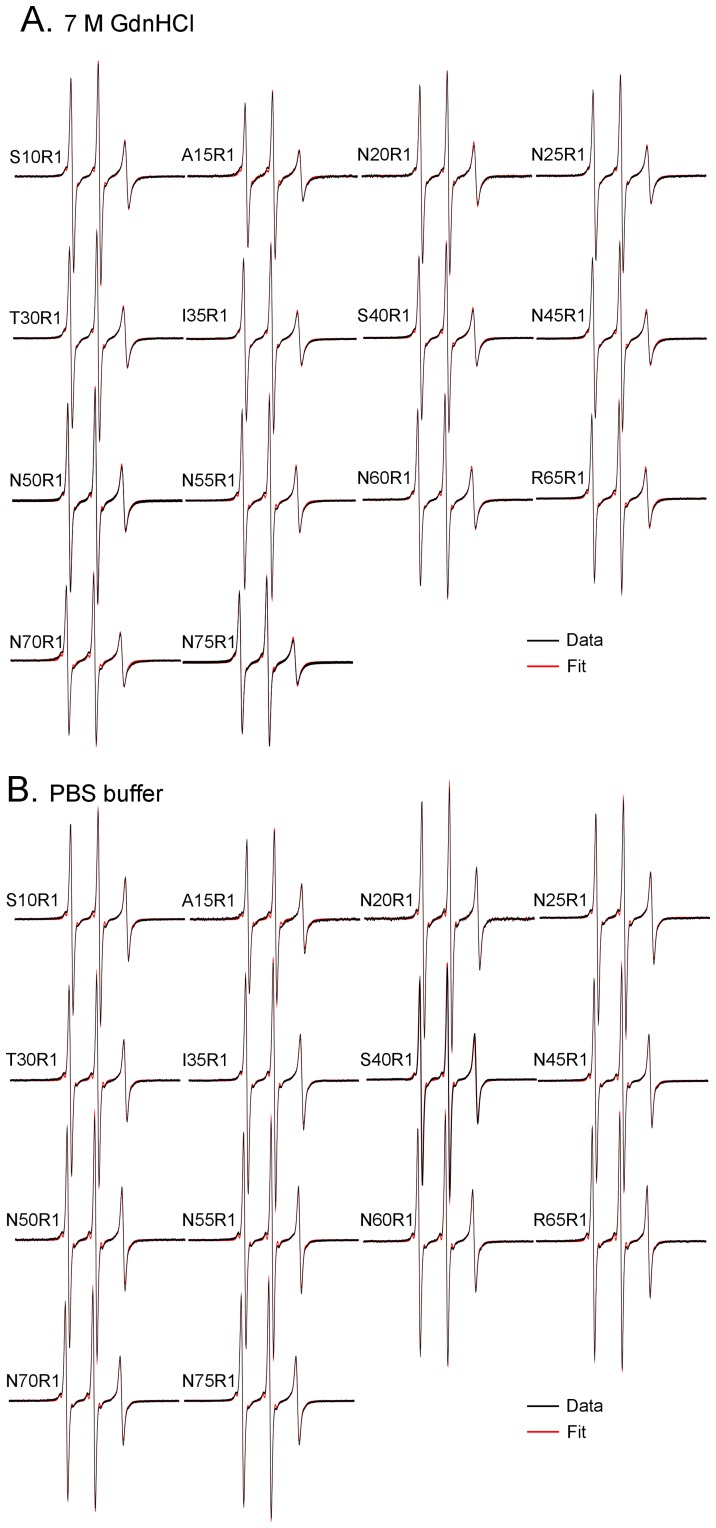
EPR spectra of spin-labeled Ure2p_1–89_-M tethered on solid support. (A) EPR spectra in 7 M GdnHCl. (B) EPR spectra in PBS. Experimental spectra are shown in black and best non-linear least squares fits are shown in red. All spectra were simulated well with one spectral component, suggesting a single structural state at all labeled sites.

Many other yeast prion proteins have also been identified [19]. All the proven yeast prion domains are rich in polar residues Q and N, and rarely contain hydrophobic and charged residues [19]. Presumably, all the yeast prion domains are intrinsically disordered [20]. Recently, an unstructured but compact state has been identified for yeast prion protein Sup35 using Förster resonance energy transfer [21], arguing that there may exist some residual structures in other Q/N-rich yeast prion proteins, including Ure2p. This has motivated us to further investigate the native structure of Ure2p prion domain using solid-support EPR.

**Figure 3:**
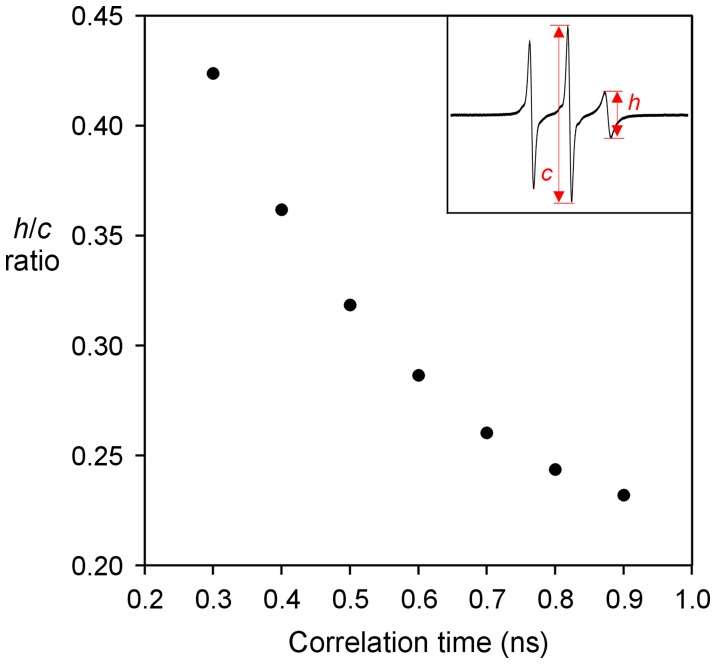
Plot of the *h/c* ratio as a function of correlation time in the range of 0.3–0.9 ns based on simulated EPR spectra. The measurements of the center and high-field line amplitude are shown in the inset. Note that the *h/c* ratio changes significantly as a function of correlation time in the sub-nanosecond range, and thus providing a sensitive measure of spin label mobility.

In this work, we have introduced a spin label named R1, one at a time, at every 5th residue from position 10 to position 75 of the Ure2p prion domain. To identify residual structures in Ure2p prion domain, we compared the spin label mobility in 7 M guanidine hydrochloride (GdnHCl) and in phosphate buffered saline (PBS). It has been suggested that most proteins under strong denaturing conditions adopt random coil structures [22]. Therefore, the Ure2p prion domain in 7 M GdnHCl serves as a reference for a completely disordered structure. Changes in spin label mobility from denaturing condition to native condition would indicate formation of ordered structures. Our results show that the site-specific residue mobility profile is similar between 7 M GdnHCl and PBS, suggesting that Ure2p prion domain is completely disordered under native conditions.

**Figure 4:**
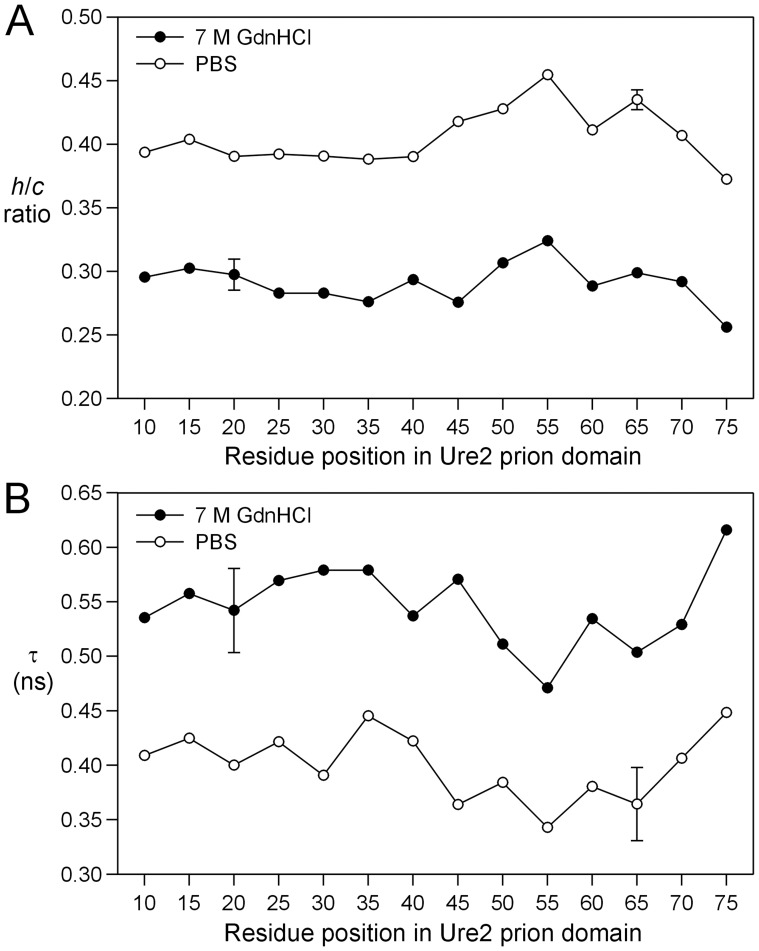
Site-specific spin label mobility in Ure2p prion domain. (A) Plot of the *h/c* ratio as a function of residue positions in Ure2p_1–89_-M. Note that the *h/c* ratio is higher in PBS, suggesting higher mobility in PBS. Additionally, the pattern of residue-specific *h/c* ratio is similar between the two conditions, suggesting similar structures. (B) Plot of correlation time from spectral simulations as a function of residue positions in Ure2p_1–89_-M. The error bars for N20R1 (in 7 M GdnHCl) and N65R1 (in PBS) are the standard deviations of two independent experiments. Other data points are results of single experiments.

## Materials and Methods

### Preparation of Ur2p Prion Domain and Spin Labeling

The construct of Ure2p prion domain was kindly provided by Dr. Susan Lindquist (Massachusetts Institute of Technology). This construct contains the Ure2p prion domain (residues 1–89) and the M domain (residues 125−253) of yeast prion protein Sup35 fused at the C-terminus [23], and is designated as Ure2p_1–89_-M. The full sequence of Ure2p_1–89_-M is shown in Figure 1. Mutants containing cysteine residue at various positions were described previously [24]. Protein expression and purification were performed as previously described [24]. Briefly, protein expression was induced with 1 mM IPTG. Cells were sonicated in a denaturing buffer containing 50 mM phosphate, 0.3 M NaCl, 8 M urea, pH 8.0 and purified with nickel column. Proteins were eluted with a linear gradient of imidazole. Protein concentration was determined by UV absorption at 280 nm using an extinction coefficient of 6.97×10^3^ M^−1^ cm^−1^ [25].

**Figure 5:**
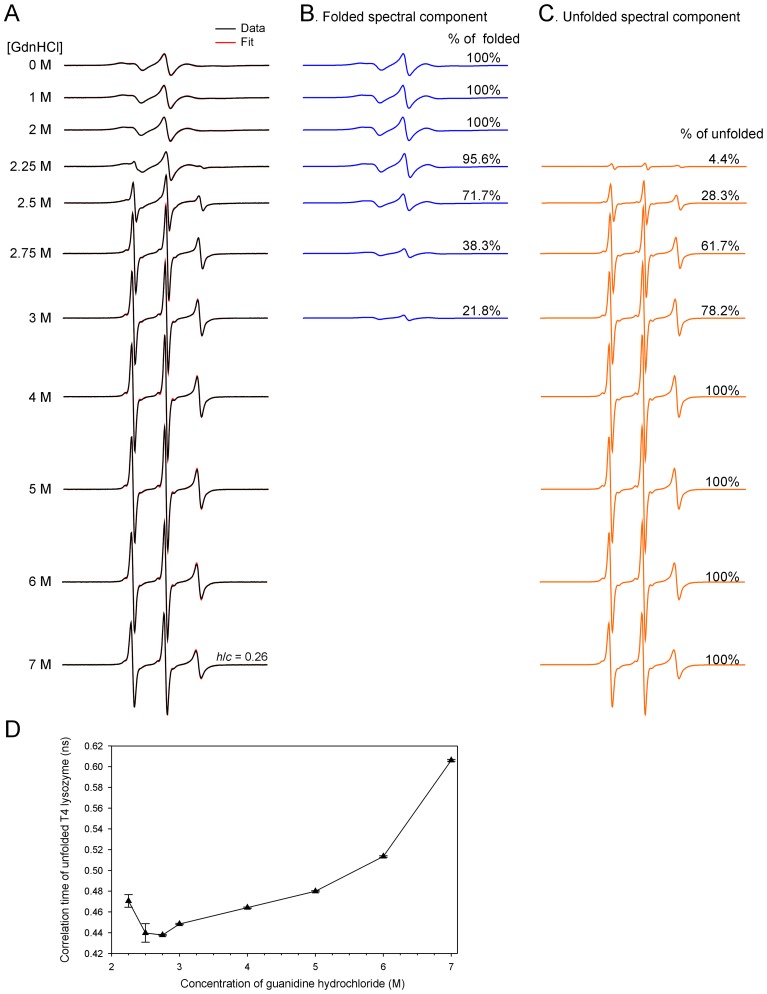
EPR studies of tethered T4 lysozyme D72R1 in denaturing conditions. (A) The EPR spectra of tethered T4L D72R1 in various concentrations of GdnHCl. The experimental spectra are shown in black and the best non-linear least squares fits are shown in red. The *h/c* ratio for the 7 M GdnHCl spectrum is calculated for comparison with those of spin-labeled Ure2p_1–89_-M. (B,C) Individual spectral components for the folded state (B) and unfolded state (C) from spectral simulations. (D) Plot of correlation time of the unfolded state from spectral simulations versus GdnHCl concentration. Note that there is a decrease in correlation time (i.e., increase in protein flexibility) when GdnHCl concentration is increased from 2.25 M to 2.75 M, suggesting that there are some residual structures in this GdnHCl concentration range. Further increase in GdnHCl concentration beyond 3 M led to a monotonic increase in correlation time, suggesting that the major effects in the GdnHCl concentration range of 3–7 M are the viscosity effect that slows down protein motion.

For spin labeling, dithiothreitol was added to protein solution to a final concentration of 10 mM and was allowed to incubate at room temperature for 20 minutes to break any disulfide bonds, and then the sample was buffer exchanged to the labeling buffer (20 mM MOPS, 8 M urea, 50 mM NaCl, pH 6.8) with a HiTrap desalting column (GE Healthcare). MTSSL, (1-oxyl-2,2,5,5-tetramethylpyrroline-3-methyl methanethiosulfonate, Enzo Life Sciences), was added at 10× molar excess immediately after dithiothreitol treatment and incubated at room temperature for 1 hour.

**Figure 6:**
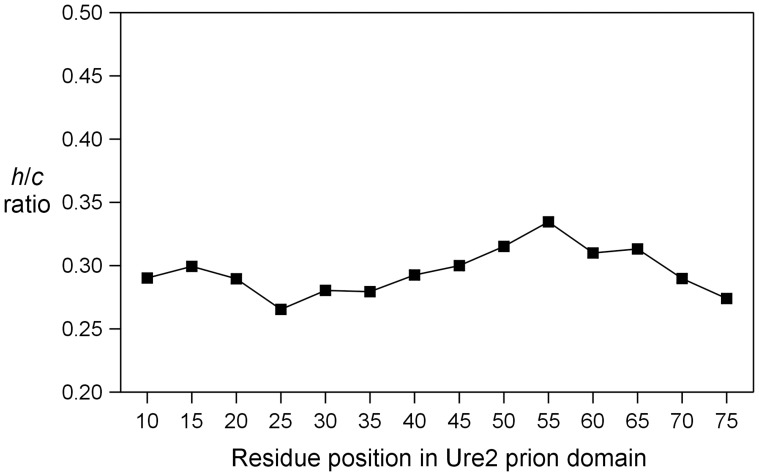
The *h/c* ratio plot for spin-labeled Ure2p_1–89_-M in PBS buffer containing 24% glycerol.

### Tethering of Ure2p Prion Domain on Solid Support

Spin-labeled proteins (typically 500 µl in volume and 50 µM in concentration) directly from labeling reaction were mixed with 100 µl of agarose beads slurry charged with nickel (Agarose Bead Technologies) and the mixture was incubated at room temperature on a nutating mixer for 1 h to allow binding. The beads were then washed three times with 500 µl of GdnHCl buffer (15 mM sodium phosphate, 7 M GdnHCl, pH 7.4). To switch to PBS or PBS with 24% (w/w) glycerol, the beads were washed three times with either PBS buffer (50 mM sodium phosphate, 140 mM NaCl, pH 7.4), or PBS-G buffer (50 mM sodium phosphate, 140 mM NaCl, pH 7.4, 24% glycerol).

**Figure 7:**
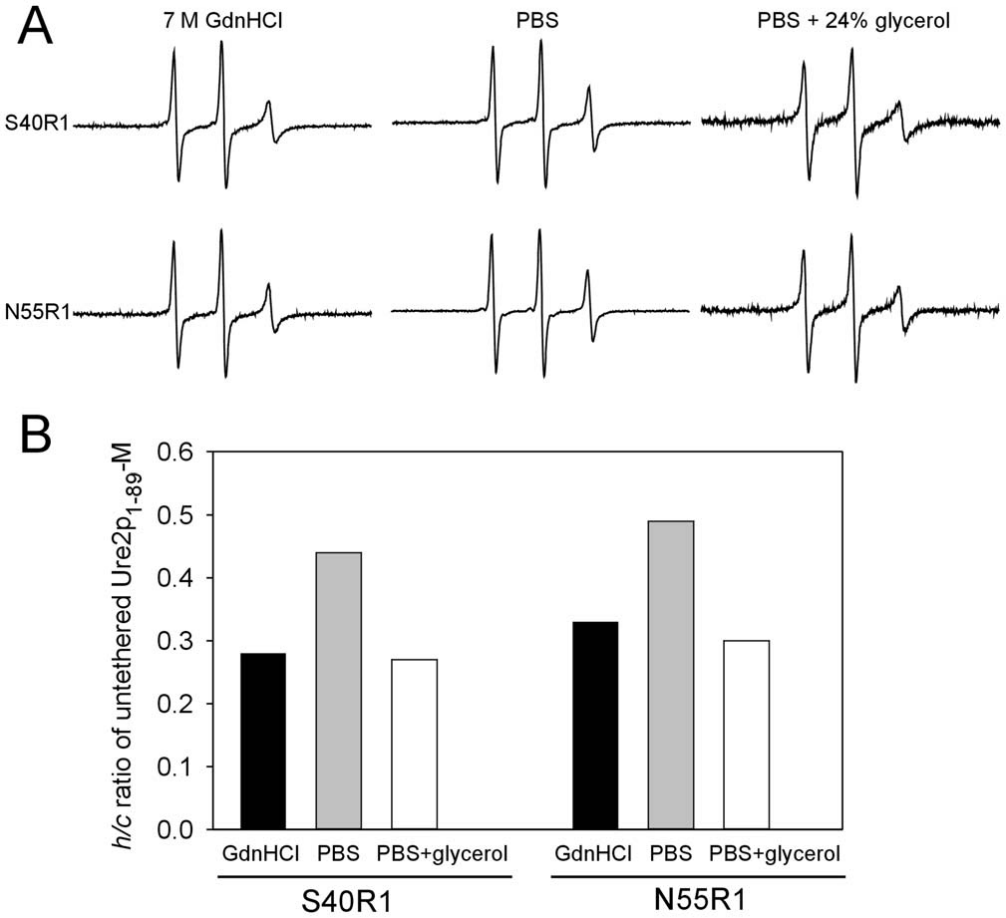
EPR studies of untethered Ure2p prion domain. (A) EPR spectra of untethered Ure2p_1–89_-M S40R1 and N55R1 in different buffer solutions. (B) The *h*/*c* ratios of untethered Ure2p_1–89_-M S40R1 and N55R1 in different buffer solutions.

### Preparation of Spin-labeled T4 Lysozyme (T4L) and Tethering on Solid Support

A D72C mutation was introduced to the cysteine-free pseudo-wild-type T4L, which contains the substitutions C54T and C97A [26], in a previous study [8]. This construct contains an N-terminal His-tag, which allows the tethering of spin-labeled T4L to nickel-charged agarose beads. Protein preparation, spin labeling, and tethering on agarose beads were performed as previously described [8]. Previously, we have performed denaturation study of tethered T4L D72R1, but only up to 5 M GdnHCl [8]. To gain insight about the effect of 7 M GdnHCl on protein mobility, the denaturation experiment was repeated with GdnHCl concentration up to 7 M. For denaturation of tethered T4L, T4L D72R1 after the spin labeling reaction was added to 200 µl of agarose beads slurry charged with nickel, and the mixture was incubated at room temperature on a nutating mixer for 1 h to allow binding of T4L onto the beads. The beads were then separated into aliquots and washed 6 times with 500 µl buffer containing 15 mM phosphate, pH 6.8, with 0–7 M GdnHCl for EPR spectroscopy.

**Figure 8:**
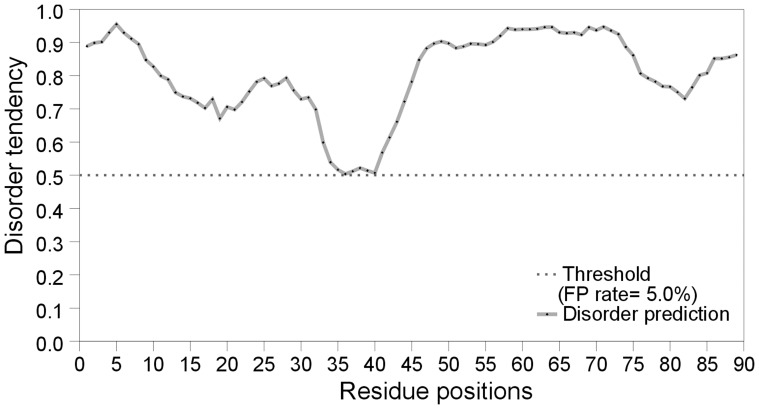
Predicted structural disorder in Ure2 prion domain using metaPrDOS [**37**]. Note that all residues are predicted to be disordered.

To evaluate if tethering on agarose beads disrupts protein structure, single cysteine mutants covering residues 114–127 of T4L were used for spin labeling and then tethered on agarose beads. These mutants have been described previously [27]. Protein preparation and spin labeling were performed as previously described [27]. These mutants are tethered on the agarose beads through their primary amine groups because they do not contain His-tags. The spin-labeled proteins were tethered on agarose beads using the AminoLink coupling gel (Pierce). The agarose beads were pre-activated to form aldehyde functional groups, which reacts with primary amines on proteins to form a secondary amine bond. Tethering was performed according to manufacturer’s instructions using a buffer containing 50 mM MOPS, 25 mM NaCl, pH 6.8.

### EPR Spectroscopy

EPR measurements were performed at X-band frequency on a Bruker EMX spectrometer equipped with the ER 4102ST cavity. A modulation frequency of 100 kHz was used. Measurements were performed at 20 mW microwave power at room temperature. Modulation amplitude was optimized to each individual spectrum (∼1 G). For each sample, approximately 20 µL of beads slurry was loaded into glass capillaries (VitroCom) sealed at one end. EPR spectra in each figure panel were normalized to the same number of spins.

### Spectral Simulations

Spectral simulations were performed using a LabVIEW (National Instruments) interface [4] of the program NLSL developed by Freed and co-workers [28], [29]. A microscopic order macroscopic disorder model was used as previously described [29]. A non-linear least squares fit of the user-defined spectral parameters was performed using the Levenberg-Marquardt algorithm. For all fits, the values for the magnetic tensor *A* and *g* were initially set as *A_xx_* = 6.2, *A_yy_* = 5.9, *A_zz_* = 37.0, and *g_xx_* = 2.0078, *g_yy_* = 2.0058, *g_zz_* = 2.0023, which were determined previously for R1 [30].

For simulation of the Ure2p_1–89_-M spectra, two parameters were allowed to vary: isotropic rotational diffusion constant (*R*), and *A*_0_ (average of *A_xx_*, *A_yy_*, and *A_zz_*). Here *A*_0_ is used as a variable parameter to account for the effect of solvent polarity on *A*_0_, but varying *A*_0_ does not affect *R*. Rotational correlation time (τ) was calculated from τ = 1/(6*R*). One and two component fits were performed. We found that using two spectral components did not significantly improve the fit, and the parameters of the two components often converge to one set of values. Therefore, we concluded that there is only one spectral component in all the simulated spectra as indicated in the text. Values of τ obtained from simulation are plotted in the figures.

For simulation of the T4L spectra, an anisotropic motional model was used for the folded state and an isotropic motion was used for the unfolded state. For anisotropic simulations, diffusion tilt angles were fixed to (α,β,γ) = (0,36°,0) for *z*-axis anisotropy as previously reported [31]. The diffusion tilt angels are the Euler angles relating the axes of the diffusion tensor and the magnetic tensor. For the folded state, *R, A*_0_, and an order parameter (*S*) were allowed to vary. For the unfolded state, *R* and *A*_0_ were used as variable parameters.

## Results

The construct of Ure2p prion domain, Ure2p_1–89_-M, contains the M domain of yeast prion protein Sup35p at the C-terminus [23]. Ure2p_1–89_-M is tethered on nickel-charged agarose beads via a His-tag at the C-terminus of the Sup35p M domain. As a result, the Sup35p M domain serves as a long linker between Ure2p prion domain and the agarose beads. Previous studies have shown that the yeast prion state [PSI+] is maintained by swapping the Sup35p Q/N-rich region with the Q/N-rich region of another yeast prion protein New1p or with a polyQ sequence, suggesting that there are no specific interactions between the Sup35p M domain and the prion domain [32]. Alberti et al [23] suggests that Sup35p M domain increases the solubility of various proven and potential yeast prion domains, allowing high level purification for *in vitro* studies. The general agreement between *in vivo* prion assays without the Sup35p M domain and *in vitro* aggregation assays with the Sup35p M domain further suggests that the Sup35p M domain has no specific interactions with various yeast prion domains, including Ure2p [23], [32]. Therefore, we assume that the attachment of the Sup35p M domain does not affect the native structure of Ure2p prion domain. We have previously shown that Ure2p_1–89_-M forms amyloid fibrils that are similar to the isolated Ure2p prion domain [24]. Structural characterization of the monomeric Ure2p_1–89_-M may reveal potential nucleating structures for the fibril formation.

To study the structure of Ure2p prion domain, we performed EPR studies of spin-labeled Ure2p_1–89_-M under two conditions: a strong denaturing condition with 7 M GdnHCl and a native condition with PBS buffer. The PBS condition is achieved by switching from 7 M GdnHCl to PBS buffer, and therefore a refolding process may have occurred. The EPR spectra in 7 M GdnHCl serve as a reference state for a completely disordered structure [22]. Our rationale is that if Ure2p_1–89_-M adopts a structured state in PBS, the structured state will lead to a decrease in the protein intrinsic dynamics on the picosecond to nanosecond timescale. Decrease in protein dynamics will then lead to decreased spin label mobility and can be detected with EPR.

Spin labels were introduced, one at a time, at every fifth residue from position 10 to position 75 (Figure 1A), covering the majority of the prion domain. A commonly used spin label R1 was used in all spin labeling studies (Figure 1B). These R1-labeled proteins were tethered on nickel-charged agarose beads. We first performed EPR studies in a strong denaturing buffer, 7 M GdnHCl. The EPR spectra are shown in Figure 2A. There are different ways to analyze spin label mobility from the EPR spectra. A commonly used measure for mobility is the inverse center line width [33]. The spectra in 7 M GdnHCl reflect very fast motion in the sub-nanosecond range, and the center line width does not provide sufficient resolution to distinguish small differences in mobility. Based on spectral simulations, the center line width increases only ∼30% when the correlation time changes from 0.3 to 0.9 ns (data not shown). Therefore, we chose to use the amplitude ratio of the high field (*h*) and the center field (*c*) lines, *h/c* ratio, as a measure of spin label mobility. The *h/c* ratio of simulated spectra changes more than 2 fold when the correlation time changes from 0.3 to 0.9 ns, providing a sensitive measure of spin label mobility in the sub-nanosecond range (Figure 3). A high *h/c* ratio corresponds to high spin label mobility, and vice versa. The calculation of *h/c* ratio does not require normalized spectra, and thus is not affected by normalization errors. Figure 4A shows the *h/c* ratio for all spin-labeled mutants of Ure2p_1–89_-M. In 7 M GdnHCl, the *h/c* ratio is between 0.25 and 0.30. For comparison, denatured T4L D72R1 has an *h/c* ratio of 0.26 in 7 M GdnHCl (Figure 5A). This is consistent with the notion that Ure2p_1–89_-M is completely disordered in 7 M GdnHCl.

The EPR spectra of tethered Ure2p_1–89_-M in native buffer (PBS) are shown in Figure 2B. The *h/c* ratio plot shows that Ure2p_1–89_-M has higher mobility in PBS than in GdnHCl (Figure 4A). This appears surprising because proteins normally adopt a more ordered state in native buffer and thus have lower mobility. In a folded protein, the spin label mobility is usually in the nanosecond range, with a corresponding *h*/*c* ratio of ∼0.2 or less. The lack of decrease in mobility when switched from GdnHCl to PBS suggests that Ure2p_1–89_-M did not form any structured states in PBS. The solution of 7 M GdnHCl has a higher viscosity than PBS buffer [34], and this may explain the lower mobility in 7 M GdnHCl. More importantly, the pattern of residue-specific mobility remains unchanged when the buffer is switched from 7 M GdnHCl to PBS (Figure 4A), suggesting that the Ure2p_1–89_-M monomer in PBS buffer adopts a similar structure as that in 7 M GdnHCl.

To provide a quantitative analysis, we performed non-linear least squares fitting to the experimental EPR spectra with spectral simulations. The spectral simulation can also overcome a potential limitation of the *h/c* ratio analysis, which does not distinguish multiple spectral components and is dominated by the more mobile component if multiple components exist. The best fits to the experimental spectra are shown as red traces in Figure 2. All the spectra were fitted well with a single spectral component, suggesting that there is only one structural state for Ure2p_1–89_-M. The rotational correlation time τ from spectral simulations is plotted in Figure 4B. Similar to the *h/c* ratio plot, the Ure2 protein showed smaller rotational correlation time in PBS than in GdnHCl, indicating faster motion in PBS. The pattern of residue-specific correlation time is similar between 7 M GdnHCl and PBS conditions, suggesting similar structures. Therefore, these results suggest that the Ure2 prion domain in Ure2p_1–89_-M adopts a completely disordered structure under native conditions.

To evaluate the effect of GdnHCl on the intrinsic protein dynamics of the denatured state, we studied the EPR spectra of T4L D72R1 in the presence of various concentrations of GdnHCl. Figure 5 shows that we can separate the spectral component for the folded state and the unfolded state using spectral simulations. Figure 5D shows that at GdnHCl concentrations of 2.25–2.75 M, increasing GdnHCl concentration resulted in a decrease in correlation time of the unfolded state, suggesting there may be residual structures at low concentrations of GdnHCl. Increasing GdnHCl concentrations from 2.25 M to 2.75 M disrupts these residual structures and thus lead to increased protein mobility. Further increase in GdnHCl concentration from 2.75 M to 7 M leads to a monotonic increase in correlation time, suggesting that the major effect of GdnHCl in this concentration range is slowing down protein motion by increasing solution viscosity. More importantly, the correlation time of the unfolded T4L is ∼0.44 ns at low concentrations of GdnHCl, and ∼0.6 ns at high concentrations of GdnHCl (Figure 5D), in agreement with the Ure2p prion domain in PBS and GdnHCl, respectively (Figure 4B). This suggests that the effect of GdnHCl on Ure2p prion domain is to simply increase solution viscosity.

To further check if the difference in spin label mobility between 7 M GdnHCl and PBS buffer can be explained by solution viscosity alone, we performed EPR studies in a PBS buffer containing 24% (w/w) glycerol, which has similar viscosity as 7 M GdnHCl [34]. Figure 6 shows that the *h*/*c* ratio in the presence of 24% glycerol is very similar to that in 7 M GdnHCl. The residue-specific pattern of *h*/*c* ratio remains largely unchanged with addition of 24% glycerol, suggesting that the overall structure is not affected by glycerol.

The disordered structure of tethered Ure2p prion domain is unlikely to be an artifact resulting from tethering on agarose beads. Previous studies have shown that tethering of proteins on a solid support generally does not perturb protein structure [35], [36]. We also showed that the EPR lineshape of T4L D72R1 remains very similar upon tethering on agarose beads [8]. To further evaluate the use of agarose beads as a solid support, we tethered 14 singly spin-labeled T4L mutants covering consecutive residues 114–127. Previous studies on these mutants without solid support showed that the spin label mobility reflects the protein topology [27]. For example, the pattern of mobility in residues 114–123 correlates well with the helical periodicity of this region [27]. Because these T4L mutants do not contain His-tags, they are tethered on agarose beads via a covalent link between primary amines on T4L and the aldehyde group on the agarose beads. Figure S1 shows that the spin label mobility of tethered T4L at every labeled residue is very similar to that of untethered T4L, confirming that agarose beads do not disrupt protein structures.

Finally, we performed EPR studies of two untethered Ure2p_1–89_-M mutants: S40R1 and N55R1. Even though Ure2p prion domain in the absence of solid support will aggregate and will give rise to more complex EPR spectra, the *h/c* ratio analysis will be dominated by the mobile spectral component, which represents the disordered structure. Figure 7A shows the EPR spectra of untethered Ure2p_1–89_-M S40R1 and N55R1. The *h/c* ratios show that the untethered Ure2p prion domain has higher mobility in PBS than in 7 M GdnHCl (Figure 7B), similar as the tethered Ure2p prion domain. With addition of 24% (w/w) glycerol, the mobility of untethered Ure2p prion domain in PBS is similar to that in 7 M GdnHCl, suggesting that the mobility difference in PBS and GdnHCl can be explained by solution viscosity alone. This suggests that the disordered structure of Ure2p prion domain did not result from interactions with agarose beads.

## Discussion

Using a solid-support EPR approach [8], we investigated the structure of isolated Ure2p prion domain in the construct of Ure2p_1–89_-M, which is tethered on solid support to prevent protein aggregation. We showed that the pattern of site-specific spin label mobility remains unchanged when the buffer is changed from a strong denaturing buffer (7 M GdnHCl) to a native buffer (PBS) after accounting for the solution viscosity (Figures 4 and 6), suggesting that the structure of Ure2p_1–89_-M in native buffer is the same as that in 7 M GdnHCl, i.e., completely disordered. Previously, we show that Alzheimer’s Aβ protein adopts both a disordered state and a structured state with solid-support EPR [8]. Therefore, we performed spectral simulations to reveal potential low mobility component in the EPR spectrum, but the results suggest that there is only one structural state for Ure2p_1–89_-M (Figure 2). Taken together, the results here show that isolated Ure2p prion domain is completely disordered without any residual structures.

The conclusion from this work is consistent with previous studies on Ure2 prion domain and with the notion that the Q/N-rich yeast prion domains are intrinsically disordered [20]. Protease digestion [16], circular dichroism measurements [17], and solution NMR [18] suggest that the Ure2p prion domain is largely disordered. Prediction of structural disorder with metaPrDOS [37], which predicts the disorder tendency of each residue by integrating results from eight independent predictors, show that all residues in the Ure2p prion domain are disordered (Figure 8). Several regions of the Ure2p prion domain have been identified to be important in the formation of amyloid fibrils. For example, residues 10–40 are the only region that is conserved among different *Saccharomyces* species [38]. Deletion of residues 15–42 lead to decreased fibril formation *in vitro* [39]. Residues 18–21 were proposed to be an amyloid stretch that initiates Ure2p fibril formation [40], [41]. A lack of ordered structure in the isolated Ure2p prion domain suggests that these regions may play a role in protein assembly rather than stabilizing local structures.

Our findings that Ure2p prion domain is completely disordered have important implications on the mechanism of its amyloid formation. Wickner and co-workers have shown that the prion domains of Ure2p and Sup35p can form prions when their primary sequences are randomly shuffled [42], [43]. Their results that the primary sequence is not important for prion formation can be rationalized by the absence of any residual structures in the prion domain. Due to the lack of any ordered structures in the monomer state, we suggest that the amyloid formation of Ure2 is driven primarily by intermolecular interactions. It has been shown that the rate of amyloid formation is determined by the competition between intramolecular and intermolecular interactions [44]. Absence of collapsed structures explains the high propensity of amyloid formation of the isolated yeast prion domains due to a lack of competition from intramolecular interactions.

## Supporting Information

Figure S1 **EPR study of tethered T4 lysozyme.** (A) Ribbon diagram of T4 lysozyme structure (PDB entry 2IGC) with the Cα atoms of residues 114–127 shown as spheres. (B) EPR spectra of tethered T4 lysozyme with spin labels introduced at indicated positions. The measurement of center line width (δ) from the EPR spectrum is shown in red. (C) Plot of inverse center line width versus residue positions. The data for untethered T4 lysozyme were published previously (Guo et al. 2007, Protein Sci. 16∶1069-86) and are reproduced here for comparison. Note that the mobility patterns for the tethered and untethered T4 lysozyme are very similar, suggesting that tethering on solid support does not disrupt T4 lysozyme structure.
(TIF)
