# Supplementary figures and images for: Prion Domain of Yeast Ure2 Protein Adopts a Completely Disordered Structure: A Solid-Support EPR Study
Source: PLoS One. 2012 Oct 16;7(10):e47248. doi: 10.1371/journal.pone.0047248 (PMC3473064; doi:10.1371/journal.pone.0047248)

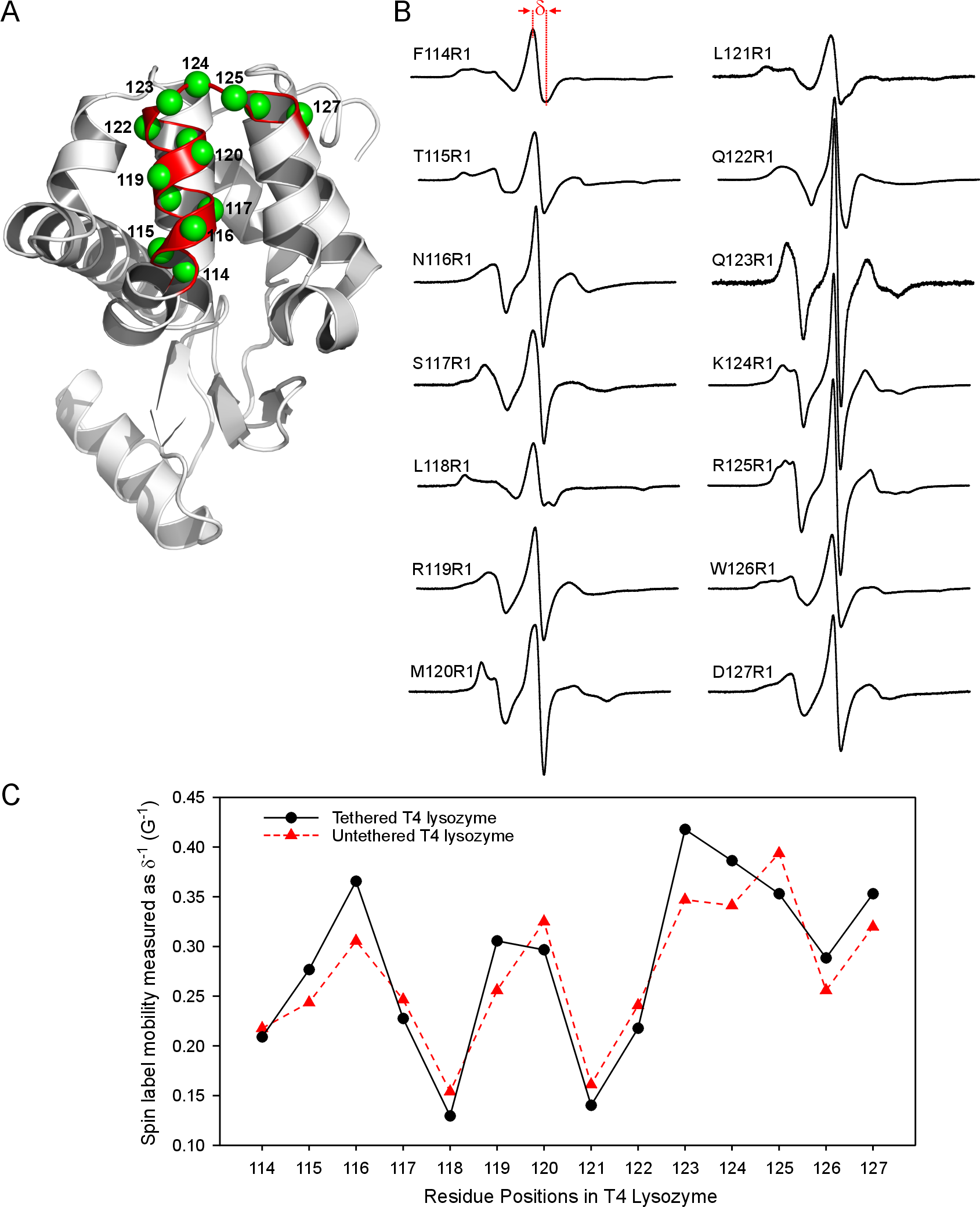

Supplement: Figure S1 — EPR study of tethered T4 lysozyme. (A) Ribbon diagram of T4 lysozyme structure (PDB entry 2IGC) with the Cα atoms of residues 114–127 shown as spheres. (B) EPR spectra of tethered T4 lysozyme with spin labels introduced at indicated positions. The measurement of center line width (δ) from the EPR spectrum is shown in red. (C) Plot of inverse center line width versus residue positions. The data for untethered T4 lysozyme were published previously (Guo et al. 2007, Protein Sci. 16∶1069-86) and are reproduced here for comparison. Note that the mobility patterns for the tethered and untethered T4 lysozyme are very similar, suggesting that tethering on solid support does not disrupt T4 lysozyme structure. (TIF) [file pone.0047248.s001.tif]
